# Identification of the Genes Chemosensitizing Hepatocellular Carcinoma Cells to Interferon-α/5-Fluorouracil and Their Clinical Significance

**DOI:** 10.1371/journal.pone.0056197

**Published:** 2013-02-15

**Authors:** Tomohiko Sakabe, Hiroyuki Tsuchiya, Keita Kanki, Junya Azumi, Kazue Gonda, Yusuke Mizuta, Daisaku Yamada, Hiroshi Wada, Kohei Shomori, Hiroaki Nagano, Goshi Shiota

**Affiliations:** 1 Division of Molecular and Genetic Medicine, Department of Genetic Medicine and Regenerative Therapeutics, Graduate School of Medicine, Tottori University, Yonago, Japan; 2 Department of Biophysical Chemistry, Kyoto Pharmaceutical University, Kyoto, Japan; 3 Department of Surgery, Graduate School of Medicine, Osaka University, Suita, Osaka, Japan; 4 Department of Microbiology and Pathology, Faculty of Medicine, Tottori University, Yonago, Japan; Johnson & Johnson Medical, China

## Abstract

The incidence of advanced hepatocellular carcinoma (HCC) is increasing worldwide, and its prognosis is extremely poor. Interferon-alpha (IFN-α)/5-fluorouracil (5-FU) therapy is reportedly effective in some HCC patients. In the present study, to improve HCC prognosis, we identified the genes that are sensitizing to these agents. The screening strategy was dependent on the concentration of ribozymes that rendered HepG2 cells resistant to 5-FU by the repeated transfection of ribozymes into the cells. After 10 cycles of transfection, which was initiated by 5,902,875 sequences of a ribozyme library, three genes including protein kinase, adenosine monophosphate (AMP)-activated, gamma 2 non-catalytic subunit (*PRKAG2*); transforming growth factor-beta receptor II (*TGFBR2*); and exostosin 1 (*EXT1*) were identified as 5-FU-sensitizing genes. Adenovirus-mediated transfer of *TGFBR2* and *EXT1* enhanced IFN-α/5-FU-induced cytotoxicity as well as 5-FU, although the overexpression of these genes in the absence of IFN-α/5-FU did not induce cell death. This effect was also observed in a tumor xenograft model. The mechanisms of *TGFBR2* and *EXT1* include activation of the TGF-β signal and induction of endoplasmic reticulum stress, resulting in apoptosis. In HCC patients treated with IFN-α/5-FU therapy, the *PRKAG2* mRNA level in HCC tissues was positively correlated with survival period, suggesting that *PRKAG2* enhances the effect of IFN-α/5-FU and serves as a prognostic marker for IFN-α/5-FU therapy. In conclusion, we identified three genes that chemosensitize the effects of 5-FU and IFN-α/5-FU on HCC cells and demonstrated that *PRKAG2* mRNA can serve as a prognostic marker for IFN-α/5-FU therapy.

## Introduction

Hepatocellular carcinoma (HCC) is the third most common cancer, the incidence of which is reportedly increasing worldwide [1]. Moreover, cases of advanced HCC, which is characterized by an extremely poor prognosis, are increasing in number. Recently, sorafenib, an oral multikinase inhibitor, was reported to improve the median overall survival rate in advanced HCC patients [2], [3]. However, it did not improve overall survival and prognosis in advanced HCC patients with portal vein tumor thrombosis (PVTT) [4]. Although interferon-alpha (IFN-α)/5-fluorouracil (5-FU) combination therapy showed favorable effects in advanced HCC patients [5], especially compared to those with PVTT [6], [7], [8], its maximum efficacy was only 25%–50% in some countries including Japan and USA, suggesting that chemoresistance limits the therapeutic potential of IFN-α and 5-FU. To achieve more favorable outcomes in advanced HCC patients, advances in IFN-α/5-FU therapy are urgently required. However, limited information is currently available on the genes involved in enhancing chemosensitivity to this therapy.

Several screening strategies using DNA microarray and RNA interference technologies are developed to identify genes with unrecognized functions. Although predictive molecular markers, including IFN-α receptor 2 [9], Wnt/β-catenin [10], and CD133 [11], are reported in association with IFN-α/5-FU therapy, little is known about the enhancing mechanisms. Therefore, we focused on ribozyme-based functional screening. Ribozymes are small catalytic RNA molecules comprising target recognition sequences and a catalytic center with RNase activity. Ribozyme libraries have been used to identify genes that are associated with several pathways [12]. Additionally, OZ1, a vector containing a ribozyme targeting the reading frames of HIV-1, was used in a clinical trial [13].

In the present study, we hypothesized that if the genes sensitizing HCC cells to 5-FU are identified, they could be applied to IFN-α/5-FU therapy and used as prognostic markers. To confirm this hypothesis, we used ribozymes to perform reverse genetics-based functional screening to identify genes that augment the efficacy of IFN-α/5-FU therapy.

## Materials and Methods

### Ethics Statement

All animal experiments were approved by the Institutional Animal Care and Use Committee of Tottori University (the permit number: 10-Y-54). The mice received humane care in accordance with the study guidelines for the care and use established by the Tottori University. All mice were kept under pathogen-free conditions and were maintained in a temperature-controlled room with a 12 h light/dark illumination cycle.

### Materials and Cell Culture

IFN-α and 5-FU were provided by MSD (Tokyo, Japan) and Kyowa Hakko Kirin Co., Ltd. (Tokyo, Japan), respectively. HepG2, HuH7, and HLF human HCC cells were obtained from the Japanese Collection of Research Bioresources and maintained in Dulbecco’s modified Eagle’s medium (Nissui Pharmaceutical Co., Ltd., Tokyo, Japan) supplemented with 10% fetal bovine serum (MBL, Nagoya, Japan), L-glutamine, and glucose in a humidified atmosphere of 5% CO_2_ at 37°C.

### The Water Soluble Tetrazolium Salt (WST) Assay

The IFN-α and 5-FU antitumor effects were assessed using the WST assay (Seikagaku Corporation, Tokyo, Japan). The cells were cultured in various concentrations of IFN-α and 5-FU for 72 h. The viability of cells treated with dimethyl sulfoxide was defined as 100%.

### Screening for Genes Involved in Enhancing the Effect of 5-FU

We constructed a plasmid DNA (pDNA) library expressing ribozyme genes (Figure S1 and Table S1). Screening was performed on the basis of the ability of 5-FU to eliminate cells expressing ribozymes that target unrelated chemosensitive genes, as described in File S1.

### Subcutaneous Xenograft Model in Mice

Four-week-old male non-obese diabetic/severe combined immunodeficient (NOD/SCID) mice were purchased from Charles River Laboratories Japan, Inc. (Kanagawa, Japan). We subcutaneously transplanted 6.8×10^6^ cells HepG2 cells in 0.1 mL PBS into the right flank of each mouse. The mice were randomly assigned to the 4 following groups; (i) *LacZ*-adenovirus and PBS (instead of IFN-α and 5-FU); (ii) *LacZ*-adenovirus and IFN-α/5-FU; (iii) *TGFBR2*-adenovirus and IFN-α/5-FU; and (iv) *EXT1*-adenovirus and IFN-α/5-FU. At 3 weeks after transplantation, 2.5×10^7^ plaque forming unit (PFU)/tumor adenoviruses carrying either the *TGFBR2*, *EXT1*, or *LacZ* genes were intratumorally injected and this was repeated every 3 d. On the next day, IFN-α and 5-FU combination treatment was started and repeated every 3 d. We administered 20,000 U/body IFN-α subcutaneously and 30 mg/kg 5-FU was administrated intraperitoneally. Administration of adenovirus carrying *TGFBR2*, *EXT1*, or *LacZ* and IFN-α/5-FU were repeated for 4 weeks according to the previous reports [14]. During the 4 weeks of treatment, tumor size was measured every week. The volumes were calculated using the following equation: (tumor volume; mm^3^) = (length; mm)×(width; mm)^2^ ×0.5^3^.

### RNA Isolation and Real-time Reverse Transcription-polymerase Chain Reaction

Total RNA from HCC cells and resected HCC specimens was extracted using TRIzol Reagent (Invitrogen, Carlsbad, CA, USA). RNA was reverse-transcribed using SuperScript II (Invitrogen) and oligo(dT) primers. mRNA expression levels were determined using the LightCycler FastStart DNA Master SYBR Green I Kit (Roche Applied Science, Basel, Switzerland) and gene-specific primers (Table S1).

### Western Blotting

Whole-cell lysates were prepared using a protease inhibitor cocktail (Roche Applied Science) and phosphatase inhibitor cocktail (Roche Applied Science). Actin was used as an internal control. The antibodies against PRKAG2 (Cell Signaling Technology, Danvers, MA, USA), TGFBR2 (Santa Cruz Biotechnology, CA, USA), EXT1 (Santa Cruz Biotechnology), SAPK/JNK (Cell Signaling Technology), Phospho-SAPK/JNK (Thr183/Tyr185) (Cell Signaling Technology), p38 MAPK (Cell Signaling Technology), Phospho-p38 MAP Kinase (Thr180/Tyr182) (Cell Signaling Technology), BAX (Santa Cruz Biotechnology), BCL-xL (Santa Cruz Biotechnology), BCL-2 (Santa Cruz Biotechnology), ATF4 (Santa Cruz Biotechnology), BiP/GRP78 (Cell Signaling Technology), CHOP (Cell Signaling Technology), LC3B (Cell Signaling Technology), and actin (Santa Cruz Biotechnology) were used in western blot analysis.

### Immunofluorescence Analysis

Immunofluorescence analysis was performed according to previous report [15]. HepG2 cells infected with adenoviruses were treated with 5-FU alone, IFN-α/5-FU or tunicamycin for 48 h. Cells were incubated with LC3B antibody (Cell Signaling Technology) followed by incubation with Alexa Fluor 488 conjugated secondary antibody (Molecular Probes, Leiden, Netherlands). DAPI was used for nuclear counterstaining.

### Patients and Clinical Specimens

Subjects included 17 multiple advanced HCC patients admitted to Osaka University Hospital between1999 and 2004, who agreed to undergo palliative reduction surgery and IFN-α/5-FU therapy after providing written informed consent. The present study was approved by the Institutional Review Board of Tottori University and Osaka University. The criteria of selection for IFN-α/5-FU therapy were based on the previous report [8]. Clinical responses of the 17 patients to IFN-α/5-FU therapy were evaluated according to the criteria of the World Health Organization [16], [17], and were classified into four categories as complete response (CR), partial response (PR), no change (NC), and progressive disease (PD). On the basis of their clinical responses, the patients were classified into the following two types: responder group (CR or PR), and non-responder group (NC or PD). Moreover, these patients were further classified into other two groups based on the presence or absence of hepatitis C virus (HCV) antibody, regardless of hepatitis B virus (HBV) infection: HCV negative group, which includes one patient with NonB/NonC and nine patients with HBV, and HCV positive group, which includes three patients with HCV and four patients with HBV/HCV.

### Statistical Analysis

For statistics, Excel spreadsheet software (Microsoft Corporation, Redmond, WA, USA) and predictive analytics software (SPSS Inc., Chicago, IL, USA) were used. Statistical comparisons were made using Student’s *t*-test, one-way analysis of variance, Tukey’s HSD test and the Mann–Whitney *U* test. The association between survival and gene expression was assessed by Spearman’s rank correlation test. *P*<0.05 was considered statistically significant.

## Results

### Identification of Protein Kinase, Adenosine Monophosphate (AMP)-activated, Gamma 2 Non-catalytic Subunit (PRKAG2); Transforming Growth Factor-beta receptor II (TGFBR2); and Exostosin 1 (EXT1) as 5-FU-sensitizing Genes

**Figure 1:**
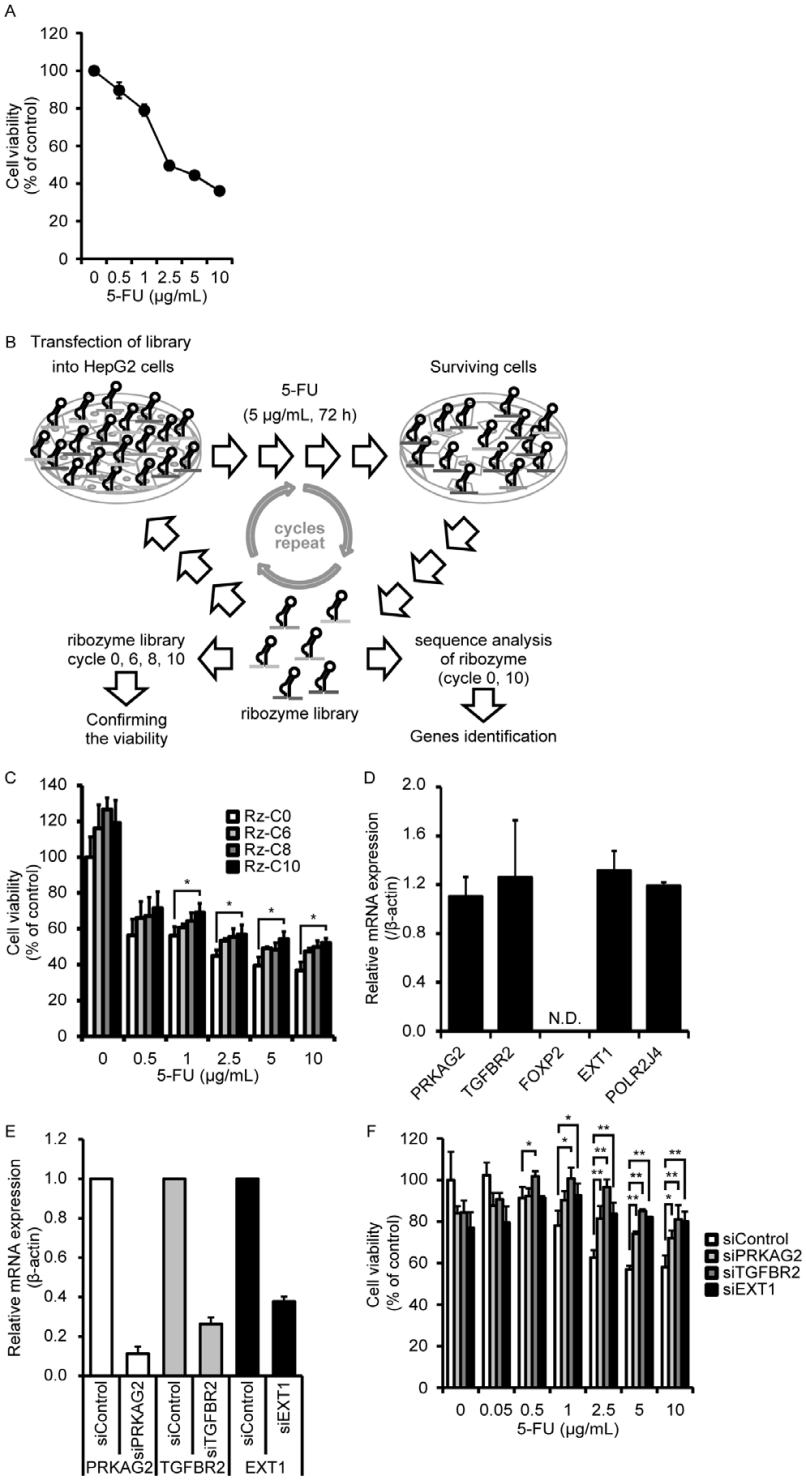
Identification of 5-FU-sensitizing genes. (A) Determination of a 5-FU concentration sufficient to decrease cell viability in HepG2 cells, which were treated with various concentrations of 5-FU for 72 h. Data are shown as means ± standard deviation (SD) (*n* = 3). (B) An outline of the screening process. The cycle was repeated 10 times, and sequence analysis of ribozymes was performed for gene identification. (C) Assessment of successful screening. HepG2 cells transfected with plasmid DNA recovered from 6 (Rz-C6), 8 (Rz-C8), and 10 (Rz-C10) cycles of screening were treated with the indicated concentrations of 5-FU for 72 h. Data are expressed as mean ± SD. Statistical significance was determined by one-way analysis of variance and Tukey’s HSD method. *P<0.05, compared to control. (D) Expression of the candidate genes in HepG2 cells. mRNA expression levels of each gene were normalized to β-actin. Data are expressed as mean ± SD (*n = *3). N.D., not detected. (E) Confirmation of the knockdown efficiencies of siRNA specific to protein kinase, adenosine monophosphate (AMP)-activated, gamma 2 non-catalytic subunit (*PRKAG2*); transforming growth factor-beta receptor II (*TGFBR2*); and exostosin 1 (*EXT1*). The expression levels of each mRNA were normalized to β-actin expression. Data are expressed as mean ± SD (*n* = 3) (F) Acquisition of resistance to 5-FU by knockdown of the identified genes. HepG2 cells were transfected with siRNA targeting *PRKAG2*, *TGFBR2*, or *EXT1*, followed by treatment with the indicated concentrations of 5-FU for 72 h. Data are expressed as mean ± SD. Statistical significance was determined using Student’s *t*-test. *P<0.05 and **P<0.01 compared to control.

We first tried to identify genes sensitizing to 5-FU instead of those sensitizing to both IFN-α and 5-FU because it seemed difficult to identify genes sensitive to both agents due to their multiple apoptotic pathways [18]–[20]. Functional screening was performed using a random ribozyme library. Briefly, HepG2 cells were first transfected with the ribozyme library containing 5,902,875 sequences, following which they were treated with 5 µg/mL of 5-FU, which is a sufficient concentration to kill HepG2 cells, for 72 h (Figure 1A). To avoid false-negative and false-positive results, we decided to adopt a moderate concentration of 5-FU for the present screening. The cells were harvested to recover ribozymes and were transfected again with the recovered ribozymes. The screening included repetition of this process for 10 rounds (Figure 1B). The cells were transfected with pDNAs that recovered from 6 (Rz-C6), 8 (Rz-C8), and 10 (Rz-C10) cycles of screening and treated with the indicated 5-FU concentrations for 72 h. The viability of cells transfected with the recovered pDNAs significantly increased during the progression from 0 to 10 cycles of screening at 1–10 µg/mL of 5-FU, suggesting that ribozymes become densely concentrated by the screening (Figure 1C). Five genes were selected as candidates because the number of ribozymes targeting the RzC10-transfected genes increased to more than four, whereas that of ribozymes targeting the Rz-C0-transfected genes was zero. (Table S2). Of the five genes, four, with the exception of FOXP2, were expressed in HepG2 cells (Figure 1D). POLR2J4 was excluded because it is a pseudogene. Since selective knockdown of the genes by specific small interfering RNA (siRNA) induced resistance to 5-FU, we subsequently focused on *PRKAG2*, *TGFBR2*, and *EXT1* (Figure 1E and 1F).

### Enhancing the Effect of the Identified Genes on IFN-α/5-FU Treatment

**Figure 2:**
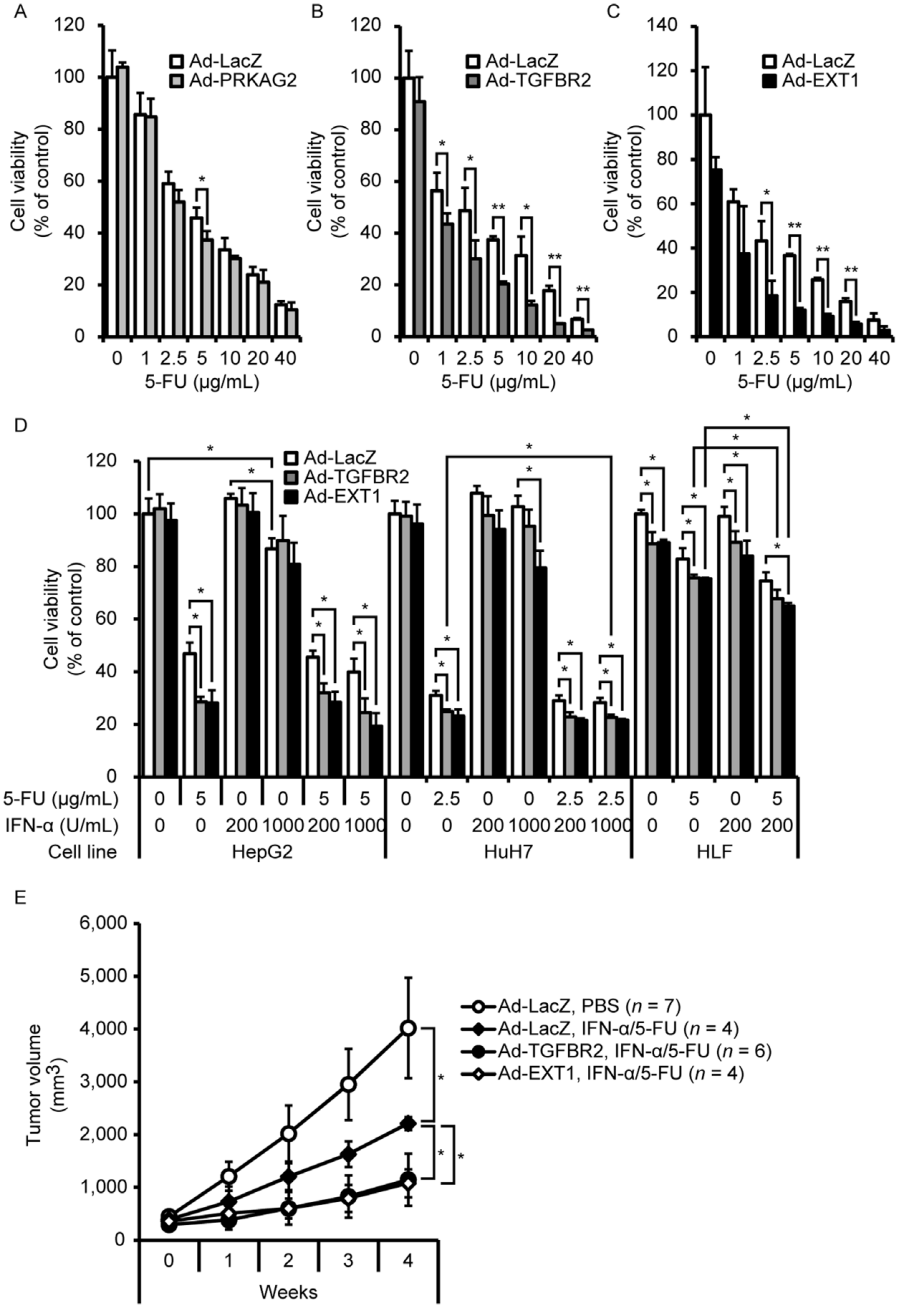
Enhancing effects of genes on IFN-α/5-FU treatment. (A–C).Viability of HepG2 cells infected with adenovirus-carrying protein kinase, adenosine monophosphate (AMP)-activated, gamma 2 non-catalytic subunit (*PRKAG2*) (A), transforming growth factor-beta receptor II (*TGFBR2*) (B), and exostosin 1 (*EXT1*) (C) with or without 5-FU. Adenovirus-carrying LacZ served as a negative control. Cell viability was determined using the WST assay at 72 h. *P<0.05 and **P<0.01, between the two groups. (D) Viability of HepG2, HuH7, and HLF cells infected with adenovirus-carrying *TGFBR2* and *EXT1* with or without agents. Data are expressed as mean ± standard deviation (SD) (*n = *3). Statistical significance was determined using Student’s *t*-test. *P<0.05 between two groups. (E) In vivo studies using NOD/SCID mice subcutaneously transplanted with HepG2 cells. In adenoviruse-administered mice, tumor growth was assessed during IFN-α/5-FU treatment. Data are shown as means ± SD (*n = *4–7).

To investigate whether these genes actually enhance 5-FU- and IFN-α/5-FU-induced cytotoxicity, *PRKAG2*, *TGFBR2*, and *EXT1* were exogenously expressed in HepG2 cells and cell viability was examined at various 5-FU concentrations (Figure 2A–C). Adenovirus-mediated expression of PRKAG2, TGFBR2, and EXT1 proteins was confirmed in these cells (Figure S2A and S2B) and mCherry fluorescent protein expression, which is used as a marker, were detected flow cytometry analysis (Figure S2C–H). Adenovirus-mediated *PRKAG2* expression enhanced the effects of 5 µg/ml of 5-FU (Figure 2A). In contrast, adenovirus-mediated *TGFBR2* expression enhanced effects at all 5-FU concentrations (1–40 µg/ml) (Figure 2B). Additionally, adenovirus-mediated *EXT1* expression accelerated the effects of 5-FU from 2.5 to 20 µg/ml (Figure 2C).

Furthermore, we focused on the effects of *TGFBR2* and *EXT1* on IFN-α/5-FU because the enhancement of *TGFBR2* and *EXT1*, but not *PRKAG2*, was effective within wide concentrations of 5-FU (Figure 2D). In HepG2 cells, 1000 U/ml of IFN-α without 5-FU was effective; however, adenovirus-mediated expression of *TGFBR2* or *EXT1* did not enhance its effects. IFN-α/5-FU greatly suppressed cell viability compared with IFN-α alone. Exogenous overexpression of *TGFBR2* and *EXT1* clearly enhanced chemosensitivity to IFN-α/5-FU as well as 5-FU. IFN-α did not kill HuH7 cells; however, adenovirus-mediated transfer of *EXT1* enhanced the effects of IFN-α, while that of *TGFBR2* also tended to be effective. The effects of IFN-α/5-FU and 5-FU were much greater than those of IFN-α alone. Moreover, adenovirus-mediated expression of *TGFBR2* and *EXT1* chemosensitized the cells to the effects of IFN-α/5-FU and 5-FU. In addition to HepG2 and HuH7 cells, *TGFBR2* and *EXT1* also enhanced the effects of 5-FU and IFN-α/5-FU in HLF cells. Then, to evaluate the significance of identified genes in IFN-α/5-FU combination treatment, we performed statistical comparisons between 5-FU alone and IFN-α/5-FU. When compared to the treatment with 2.5 µg/mL 5-FU alone, a combined effect of 200 U/mL IFN-α and 2.5 µg/mL 5-FU was observed in *TGFBR2*-overexpressing HuH7. Similarly, 200 U/mL IFN-α and 5 µg/mL 5-FU showed statistical significance in *TGFBR2*- or *EXT1*-overexpressing HLF cells.

Additionally, *in vivo* studies were performed using NOD/SCID mice subcutaneously transplanted with HepG2 cells. In mice administered adenoviruses expressing either *TGFBR2* or *EXT1*, tumor growth suppression by IFN-α/5-FU was significantly enhanced compared with that in controls (Figure 2E). These findings suggest that overexpression of TGFBR2 and EXT1 enhance chemosensitivity to IFN-α/5-FU *in vitro* and *in vivo* and support our hypothesis that if the genes sensitizing HCC cells to 5-FU are identified, they could be applied to IFN-α/5-FU therapy.

### Molecular Mechanisms Underlying the Enhancement of Antitumor Effects by TGFBR2 and EXT1

**Figure 3:**
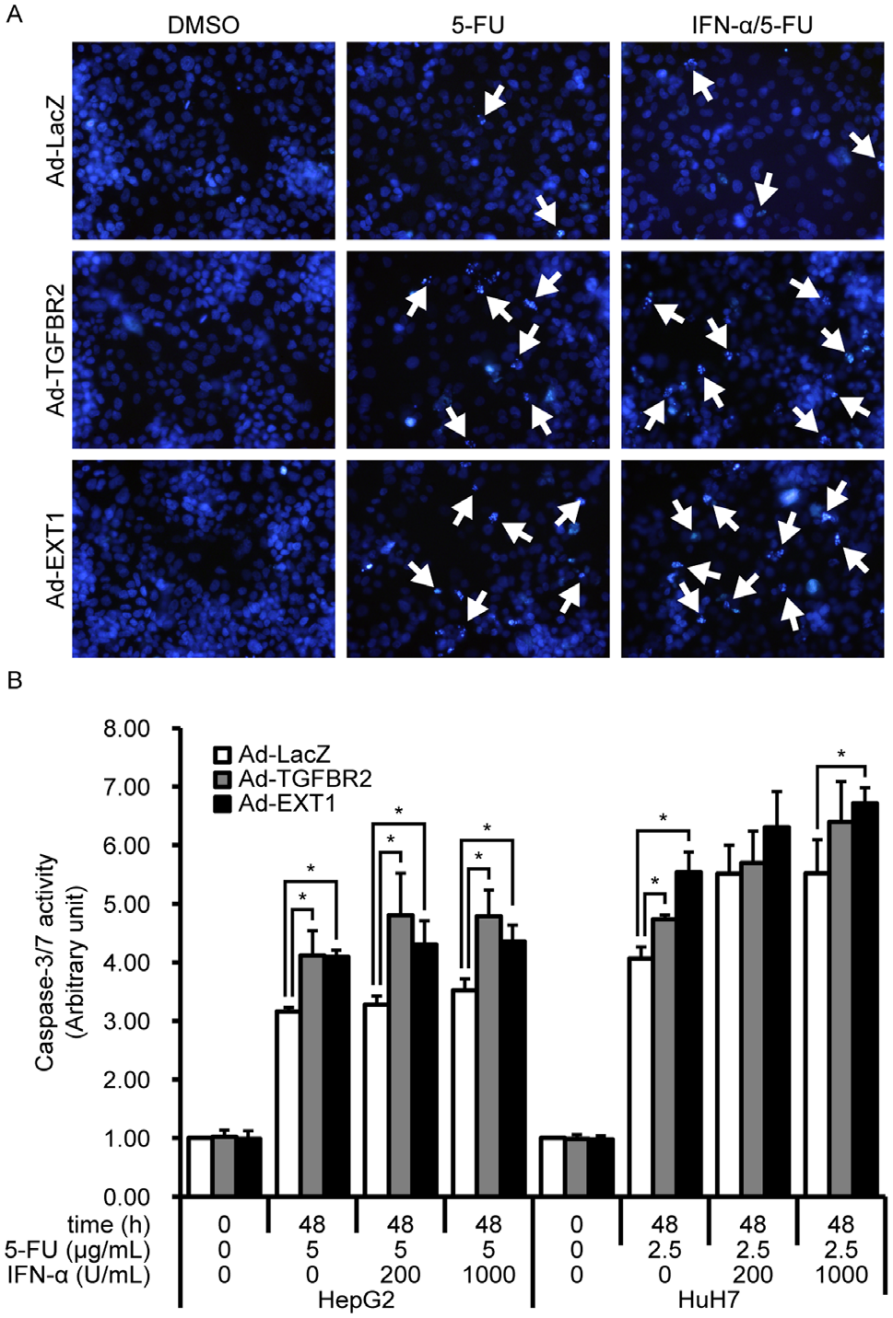
Enhancement of IFN-α/5-FU-induced apoptosis by *TGFBR2* and *EXT1* overexpression. (A) Evaluation of nuclear condensation. The arrows indicate cells with apoptosis-specific nuclear condensation and fragmentation. (B) Measurement of intracellular caspase 3/7 activity. After adenovirus infection, cells were treated with the indicated concentrations of IFN-α and 5-FU. Activity was expressed as the fold increase relative to that at 0 h. Data are expressed as mean ± standard deviation (*n = *3). Statistical significance was determined using Student’s *t*-test. *P<0.05 compared to LacZ at 48 h.

Although the genes greatly enhanced the antitumor effects of IFN-α/5-FU, the underlying mechanisms remained unclear. The principal mechanism underlying the antitumor effects of IFN-α/5-FU or 5-FU alone in several HCC cells is apoptosis [21]. First, we examined whether *TGFBR2* and *EXT1* enhanced apoptosis induced by IFN-α/5-FU. As reported, nuclear condensation of HepG2 cells was induced by 5-FU and IFN-α/5-FU (Figure 3A and S3). Adenovirus-mediated expression of *TGFBR2* and *EXT1* increased nuclear condensation compared with the expression of *LacZ* (Figure 3A and S3). Supporting this observation, caspase 3/7 activity was increased by 5-FU and IFN-α/5-FU treatment in HepG2 and HuH7 cells, respectively (Figure 3B). Further enhancement of caspase 3/7 activation was indicated by overexpression of *TGFBR2* and *EXT1* in these cells. These results suggest that *TGFBR2* and *EXT1* enhanced IFN-α/5-FU effects by accelerating apoptosis.

### Regulation of Apoptosis by TGFBR2

**Figure 4:**
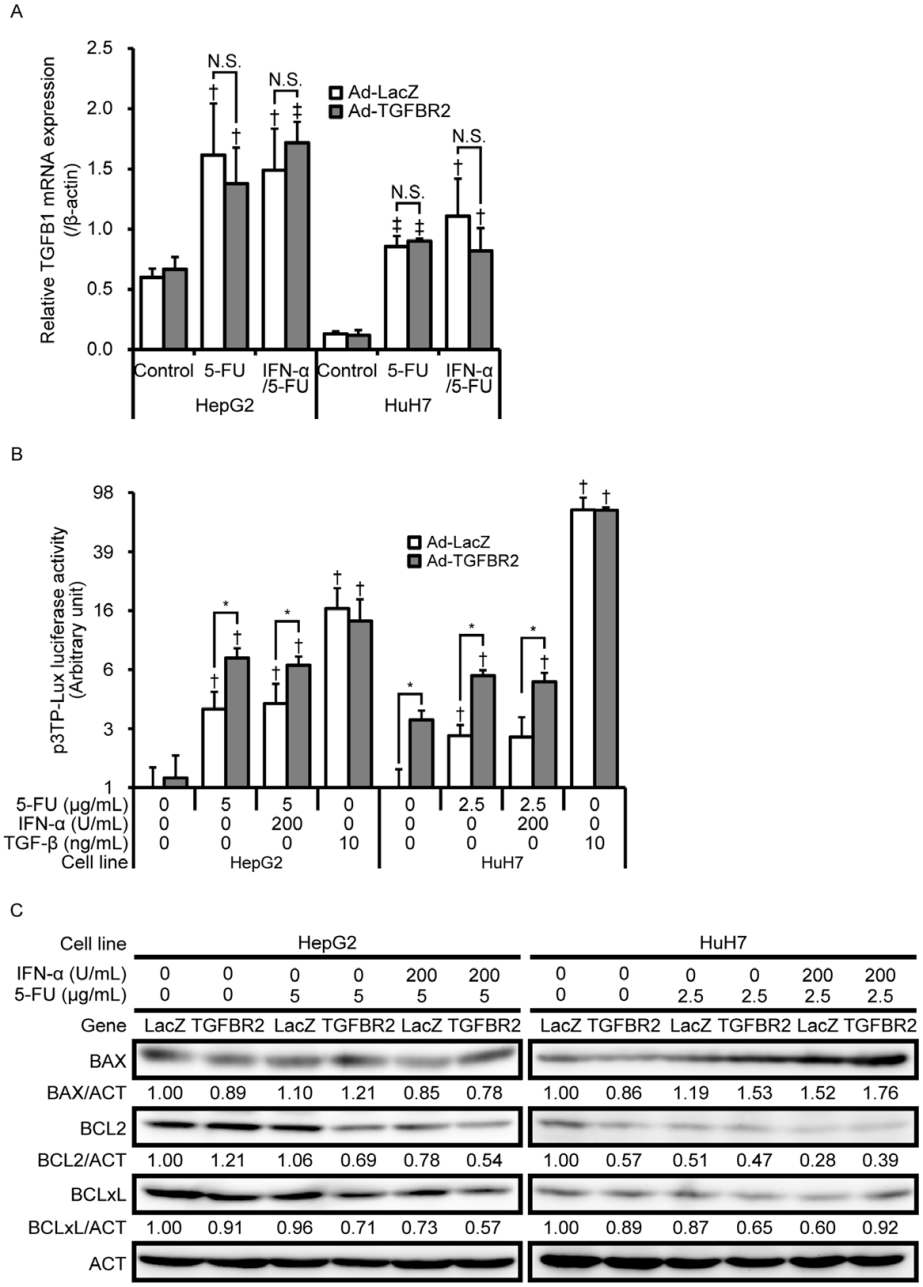
Effects of TGFBR2 on 5-FU- and IFN-α/5-FU-induced TGF-β signaling pathway. (A) 5-FU-induced *TGFB1* mRNA expression normalized to β-actin. Data are expressed as mean ± standard deviation (SD) (*n = *3). Statistical significance was determined using Student’s *t*-test. †*P*<0.05 and ‡*P*<0.001 compared to control. N.S., not significant. (B) Luciferase reporter assay for TGF-β signaling activation. Data are expressed as mean ± SD (*n = *3). Recombinant TGF-β was used as a positive control. The Y-axis is expressed as a logarithmic scale. (C) Western blot analysis of BAX, BCL-2, and BCL-xL in 5-FU and IFN-α/5-FU treatment with or without *TGFBR2* overexpression. Each band was quantified by Image J software and normalized to actin.

To explore the precise role of *TGFBR2* in enhancing apoptosis by IFN-α/5-FU, we investigated possible mechanisms. First, we determined that *TGFB1* mRNA levels after 5-FU and IFN-α/5-FU treatment were significantly increased, although neither *TGFBR2* nor *LacZ* affected their expression (Figure 4A). The reporter assay using TGF-β-responsive plasmids showed that 5-FU and IFN-α/5-FU induced TGF-β-dependent transcriptional activity in HepG2 and HuH7 cells (Figure 4B). Additionally, *TGFBR2* overexpression significantly enhanced TGF-β signaling compared with that in the controls. These findings suggest that the TGF-β signaling pathway is involved in the antitumor mechanisms of 5-FU alone or IFN-α/5-FU, and that *TGFBR2* overexpression facilitates its autocrine stimulation. Next, we examined the protein levels of proapoptotic BAX and antiapoptotic BCL-2 and BCL-xL, which are targets of the TGF-β signaling pathway during apoptosis induction [22]–[24]. In HepG2 cells, *TGFBR2* overexpression induced BAX expression in the presence of 5-FU and IFN-α/5-FU (Figure 4C). Moreover, 5-FU alone did not alter BCL-2 levels, whereas IFN-α/5-FU decreased BCL-2 levels, which were further decreased by *TGFBR2* overexpression. However, only a slight change in BCL-xL levels was observed after 5-FU or IFN-α/5-FU treatment or with *TGFBR2* expression. In HuH7 cells, 5-FU and IFN-α/5-FU induced BAX expression, which was further induced by exogenous *TGFBR2* expression, whereas BCL-2 and BCL-xL were only slightly affected. Additionally, it was reported that TGF-β also induces apoptosis by activating the c-jun N-terminal kinase (JNK) and p38 mitogen-activated protein kinase (MAPK) signaling pathway in a Smad-independent manner [25]–[28]. Therefore, we examined their phosphorylation by western blot analysis. 5-FU and INF-α/5-FU treatment increased the phosphorylation of JNK, but not of p38, in HepG2 and HuH7 cells (Figure S4). Although, *TGFBR2* overexpression increased phosphorylation of JNK in HepG2 cells in the absence of 5-FU or IFN-α/5-FU, the cell viability was not altered as indicated in Figure 2B. Additionally, *TGFBR2* overexpression did not altered the phosphorylation of JNK and p38 in the presence of 5-FU or IFN-α/5-FU, indicating that the MAPK signaling pathway is unlikely involved in the enhancing mechanisms of *TGFBR2* overexpression (Figure S4). Taken together, these results indicate that *TGFBR2* overexpression activates the Smad-dependent TGF-β signaling pathway and modulates the expression of apoptosis-related genes, including *BAX*, *BCL-2*, and *BCL-xL*.

### Induction of Endoplasmic Reticulum (ER) Stress by EXT1

**Figure 5:**
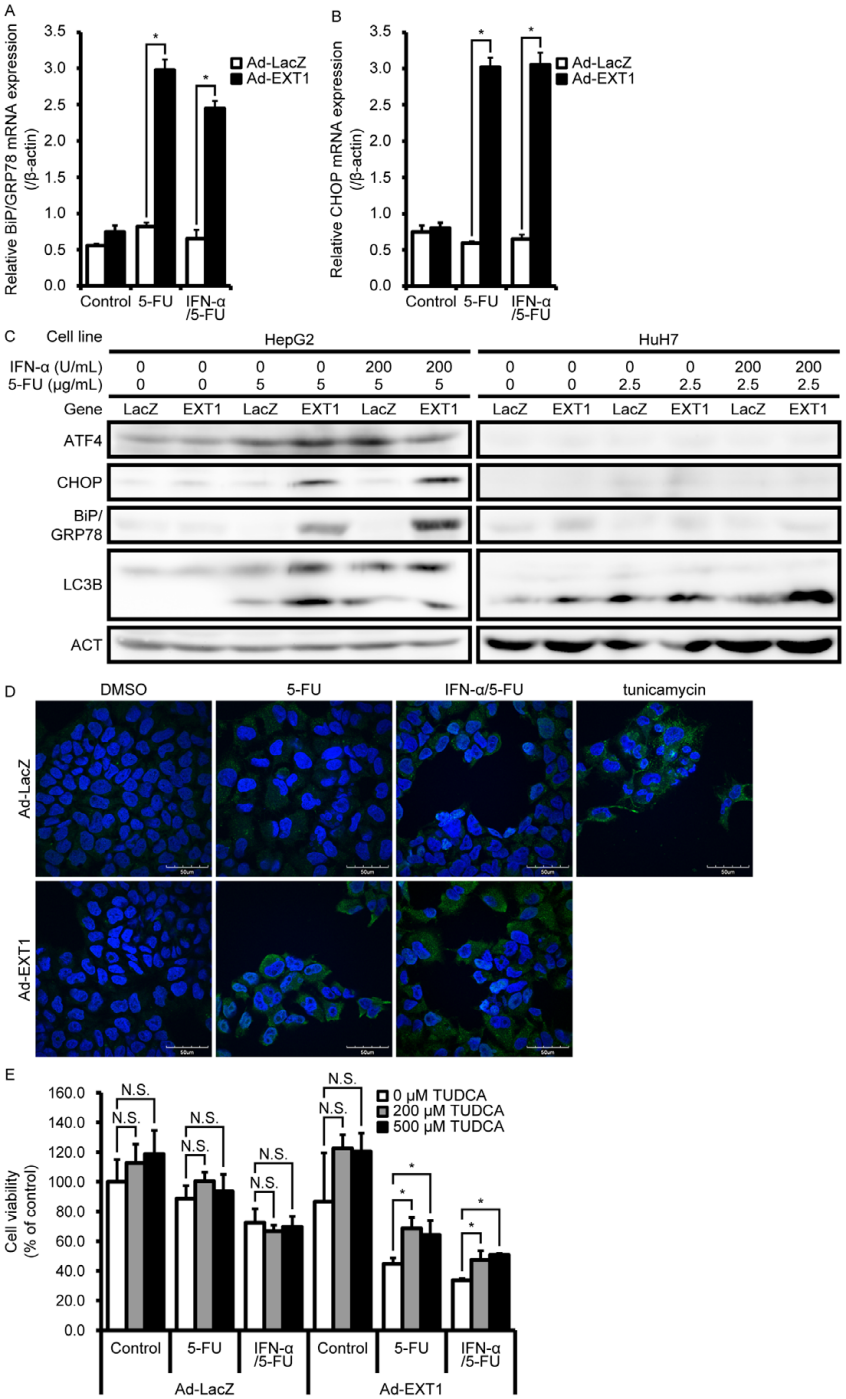
Effect of *EXT1* expression on ER stress with or without 5-FU and IFN-α/5-FU. (A, B) Real-time reverse transcription-polymerase chain reaction analysis of *BiP/GRP78* (A) and *CHOP* (B) mRNA expression. mRNA expression levels were normalized to β-actin. Data are expresses as mean ± standard deviation (*n* = 3). Statistical significance was determined using Student’s *t*-test. *P<0.05, compared to control. (C) Western blot analysis for ATF4, CHOP, BiP/GRP78, and LC3B. Actin was used as an internal control. (D) LC3B expression in HepG2 cells treated with 5-FU in the presence and absence of IFN-α. Cells infected with adenoviruses were treated with 5 µg/mL of 5-FU alone and in combination with 200 U/mL of IFN-α for 48 h. LC3B (green) and nuclei (DAPI, blue) signals were examined by immunofluorescence analysis using confocal microscopy. Treatment with 20 mg/mL of tunicamycin was used as a positive control. (E) Viability of HepG2 cells infected with adenovirus-carrying EXT1 in the presence or absence of TUDCA. Cells were treated with 5 µg/mL of 5-FU alone and in combination with 200 U/mL of IFN-α for 72 h. During this treatment, cells were exposed to TUDCA. Data are expressed as mean ± standard deviation (SD) (*n = *3). Statistical significance was determined using Student’s *t*-test. *P<0.05 between two groups. N.S., not significant.

*EXT1* overexpression enhanced IFN-α/5-FU-induced apoptosis; however, the relationship between *EXT1* and apoptosis remains unclear. *EXT1* encodes an ER-resident type II transmembrane glycosyltransferase that is involved in the chain elongation step of heparan sulfate biosynthesis [29]. Because the link between ER stress and apoptosis has been established [30], [31], and because EXT1 is predominantly localized in the ER [29], we examined whether *EXT1* induces ER stress. In HepG2 cells, *EXT1* overexpression in the presence of 5-FU and IFN-α/5-FU induced significant upregulation of *BiP/GRP78* mRNA (Figure 5A). Similarly, a significant elevation of *CHOP*, a hallmark of ER stress-induced apoptosis [30], [31], was also observed (Figure 5B). ER stress triggers ATF4 translation [30]. As shown in Figure 5C, along with BiP/GRP78 and CHOP expression, *EXT1* further enhanced 5-FU-induced ATF4 expression. Meanwhile, in HuH7 cells, these ER stress markers were undetectable by 5-FU, IFN-α/5-FU, or *EXT1* overexpression (Figure 5C). Furthermore, ER stress is closely linked to autophagy, which is a physiological response similar to apoptosis [32]. As shown in Figure 5C, the conversion of LC3B-I to LC3B-II (markers of autophagy) was significantly increased by 5-FU and IFN-α/5-FU treatment and particularly enhanced by *EXT1* overexpression in HepG2 and HuH7 cells. Similar findings were observed by immunofluorescence analysis with an LC3B antibody (Figure 5D). To investigated whether the enhancing effect of *EXT1* on IFN-α/5-FU treatment is involved in ER stress. To this end, we examined the effect of tauroursodeoxycholate (TUDCA), which is a chemical chaperone that ameliorates ER stress [5]–[7], on the viability of the cells treated with 5-FU alone or IFN-α/5-FU [33]–[35]. As shown in Figure 5E, TUDCA treatment recovered the cell viability of *EXT1*-overexpressing HepG2 cells in the presence of 5-FU alone or IFN-α/5-FU, whereas it was not observed in *LacZ*-overexpressing HepG2 cells, indicating that increased ER stress is involved in the mechanisms by which *EXT1* enhances the cytotoxic effect of 5-FU and IFN-α/5-FU. These data suggest that *EXT1* overexpression enhanced the effects of IFN-α/5-FU through ER stress-induced autophagy and apoptosis.

### Association of PRKAG2 and TGFBR2 Expression with the Clinical Outcomes of IFN-α/5-FU Therapy in Advanced HCC Patients

**Figure 6:**
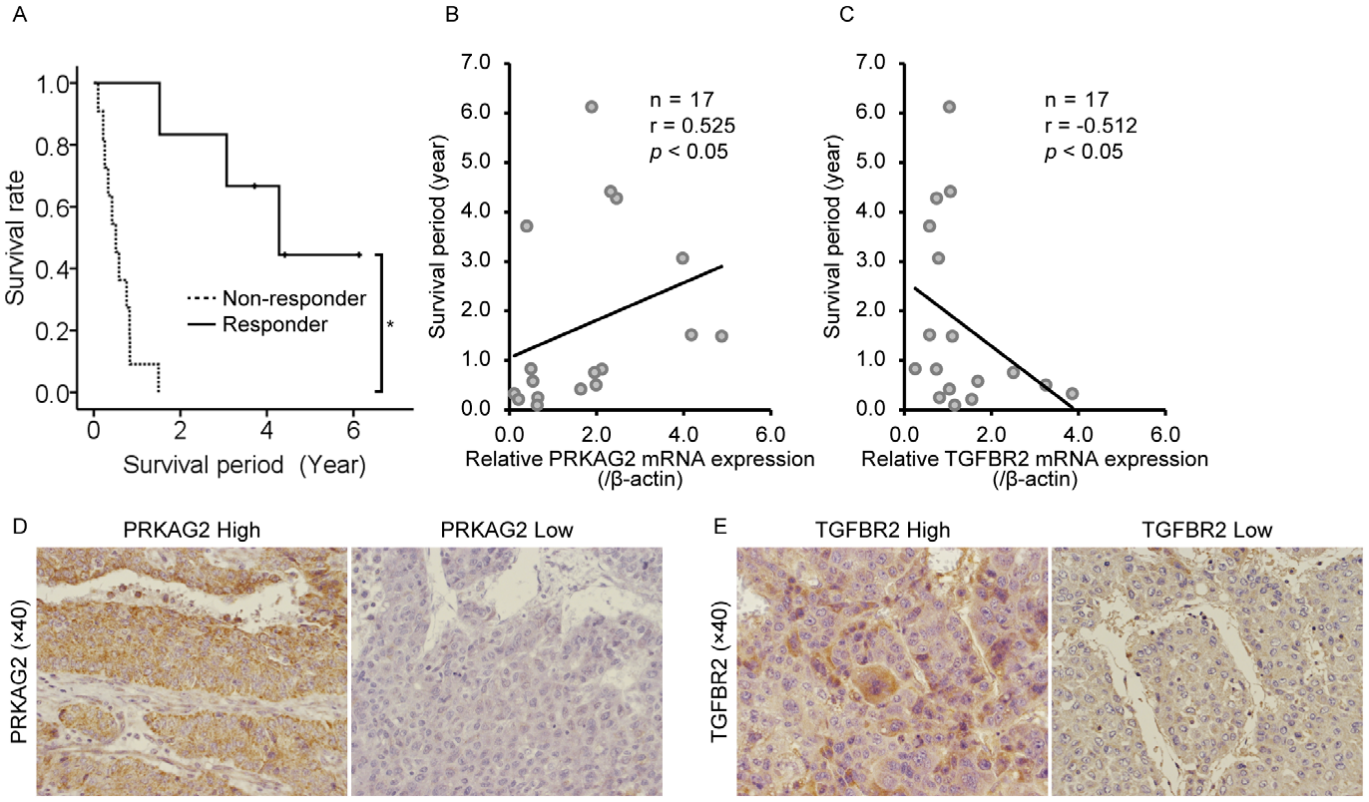
The correlation between gene expression levels and response to IFN-α/5-FU therapy. (A) Survival rates of responders and nonresponders treated with IFN-α/5-FU. *P<0.05 between groups. (B) The correlation between protein kinase, adenosine monophosphate (AMP)-activated, gamma 2 non-catalytic subunit (*PRKAG2*) mRNA and survival period in patients treated with IFN-α/5-FU therapy. Data were analyzed using Spearman’s rank correlation test. (C) The correlation between transforming growth factor-beta receptor II (*TGFBR2*) mRNA and survival period in patients treated with IFN-α/5-FU therapy. Data were analyzed using Spearman’s rank correlation test. (D, E) Immunohistochemical analysis of PRKAG2 (D) and TGFBR2 (E) expression.

To gain further insight into the clinical significance of these genes, we examined the relationship between their mRNA expression levels in tumor tissues and clinical outcomes in 17 advanced HCC patients treated with IFN-α/5-FU therapy. The clinical parameters of the patients are summarized in Table 1. Responders to IFN-α/5-FU therapy survived longer than non-responders (*P*<0.05, Figure 6A), suggesting that patients sensitive to IFN-α/5-FU therapy can achieve longer overall survival. *PRKAG2* expression tended to be higher in responders than in non-responders (Figure S5A) and was positively correlated with survival period (*P*<0.05, Figure 6B), indicating that *PRKAG2* expression can serve as a prognostic marker for IFN-α/5-FU therapy. In sharp contrast to *PRKAG2*, *TGFBR2* expression was significantly lower in responders than that in non-responders (Figure S5B) and negatively correlated with the survival period (*P*<0.05, Figure 6C). *EXT1* expression showed no difference between responders and non-responders with no correlation between *EXT1* expression and survival period (Figure S5C and S5D). The association between immunohistochemical analyses and survival in four representative cases are described as follows. A 51-year-old female patient (no. 07642160) with high *PRKAG2* expression in tumor tissue survived for 1.519 years (Figure 6D, left panel), whereas a 32-year-old male patient (no.08258681) with low *PRKAG2* expression survived for 0.331 years (Figure 6D, right panel). A 53-year-old male patient (no.08205481) with high *TGFBR2* expression survived for 0.508 years (Figure 6E, left panel), whereas a 53-year-old male patient (no.08492686) with low *TGFBR2* expression survived for 3.714 years (Figure 6E, right panel).

**Table 1:** Clinical parameters of HCC patients.

No of patients	17
Age (years)	56±12
Gender	
Male/Female	16/1
Child-pugh	
A/B/C	11/6/0
Etiology	
HBV	9
HCV	3
HBV/HCV	4
NonB/NonC	1
HCV antibody	
Negative/Positive	10/7
Tumor size (cm)	10.09±5.10
PVTT	
Vp3/Vp4	4/13
Survival period (year)	1.73 (0.1–6.13)
Response (WHO)	
CR/PR/NC/PD	3/3/1/10

Parenthetical values in survival period showed minimum and maximum survival days.

Additionally, univariate analysis revealed that factors associated with the survival of patients treated with IFN-α/5-FU therapy included hepatitis C virus (HCV) infection and *PRKAG2* mRNA levels (Table S3), although the number of patients was small. HCC patients positive for anti-HCV antibodies survived longer than those testing negative for these antibodies (*P*<0.05, Figure S5E), as previously reported [36]. HCV-positive patients had lower *TGFBR2* levels compared with HCV-negative patients (*P*<0.05, Figure S5F).

## Discussion

Based on our hypothesis, we successfully identified genes that enhanced 5-FU- and IFN-α/5-FU-induced cytotoxicity of HCC cells when overexpressed *in vitro*. The identified genes, especially *PRKAG2* and *TGFBR2*, can serve as prognostic markers for IFN-α/5-FU therapy.

*PRKAG2* encodes the gamma 2 non-catalytic but regulatory subunit of AMP-activated protein kinase (AMPK), an important cellular homeostasis sensor. Activated AMPK reserves cellular energy content and serves as the key determinant of cell survival in response to pathological energetic, oxidative, and ER stress [37]. Indeed, reports documenting that AMPK significantly enhances 5-FU antitumor effects via COX-2 suppression and inhibits the mTOR signaling pathway that regulates tumor growth may support our observations [38], [39]. We found that siRNA-mediated knockdown of *PRKAG2* strongly inhibited chemosensitivity to 5-FU; however, the effects of adenovirus-mediated overexpression of *PRKAG2* on 5-FU were small compared with those of adenovirus-mediated overexpression of *TGFBR2* or *EXT1*. These results can be explained as follows. *PRKAG2* overexpression without the other subunits may be stoichiometrically insufficient to activate the AMPK complex, while its knockdown may be sufficient to suppress its kinase activity. In the present study, *PRKAG2* expression was positively correlated with survival period in HCC patients treated with IFN-α/5-FU. Indeed, phosphorylated AMPK expression has been associated with survival period and disease-free survival in HCC and lung cancer patients [40], [41]. Taken together, AMPK activation may inhibit tumorigenesis.

TGFBR2 is a type II TGF-β receptor that, upon binding to its ligand, triggers various responses, including proliferation, differentiation, and apoptosis [42]. In the present study, *TGFB1* mRNA was not increased by the infection with Ad-TGFBR2. This result could be explained as follows: Because TGF-β is a pleiotropic cytokine, the drug-induced *TGFB1* expression may provide an advantage for cancer cell survival, possibly in an endocrine fashion *in vivo*, such as immunosuppression and angiogenesis [43], [44]. Therefore, our observation suggests that *TGFBR2* expression level may be a critical factor to determine the fate of the role of TGF-β in cancer cells. However, the discrepancy between the increased chemosensitization to IFN-α/5-FU *in vitro* by *TGFBR2* and the negative correlation of *TGFBR2* with survival period in patients treated with IFN-α/5-FU remains unclear. The TGF-β signaling pathway reportedly exhibits paradoxical roles of tumor suppression and oncogenesis. It is well known that TGF-β signaling is a potent suppressor of HCC cells [45]. A previous report [46] showed that TGF-β enhanced the lethal effects of 5-FU in human lung cancer cells; this supports our *in vitro* data that IFN-α/5-FU-induced apoptosis was enhanced by *TGFBR2* expression. On the contrary, altered TGF-β signaling reportedly plays an important role in HCC progression [47], [48]. Indeed, the TGF-β signaling pathway promotes hepatocarcinogenesis in experimental p53-depleted mice [49]. Furthermore, epithelial–mesenchymal transition (EMT), the underlying molecular mechanisms of which include the TGF-β pathway, is increasingly being recognized to occur during HCC progression [50]. These reports and our findings suggest that TGF-β signaling may be associated with tumor progression or development in patients with advanced HCC, rather than enhancing the antitumor effect of IFN-α/5-FU. Additionally, downregulation of *TGFBR2* mRNA has been reported in HepG2 cells transfected with an HCV clone [51], suggesting that a potential benefit of HCV infection may exist. In consistent with this notion, a significantly prolonged survival period during IFN-α/5-FU therapy was observed in patients infected with HCV. Further evaluations are required to determine these associations between HCV infection, *TGFBR2* expression, and EMT.

EXT1 expression was reported to be epigenetically silenced in tumors, while the restoration of EXT1 expression in cancer cells induced tumor-suppressive effects [52]. However, to our knowledge, little is known about the relationship between *EXT1* expression and HCC. In our present study, *EXT1* overexpression induced ER stress in HepG2 cells in the presence of 5-FU and IFN-α/5-FU. Induction of ER stress by *EXT1* overexpression through the reduction of heparin sulfate N-sulfation has been reported [53]. As per these observations, *EXT1* may sensitize HCC cells to 5-FU through ER stress, which is induced by alternating heparin sulfate posttranslational modification.

In conclusion, we identified *PRKAG2*, *TGFBR2*, and *EXT1* as chemosensitizing genes of HCC cells to 5-FU. Furthermore, *TGFBR2* and *EXT1* overexpression enhanced the anti-tumor effects of IFN-α/5-FU on HCC cells. These genes are promising candidates to enhance the therapeutic effects of IFN-α/5-FU.

## Supporting Information

Figure S1 **Scheme for construction of ribozyme library.** A plasmid DNA (pDNA) library expressing random ribozyme genes with as large as about 6×10^6^ target recognition sequences from synthesized oligonucleotides (Rz1–Rz6; the sequences were listed in Table S1) using PCR and the Gateway technologies.
(TIF)

Figure S2 **Detection of adenovirus-mediated gene transfer.** These protein expressions after adenovirus-mediated gene transfer were examined in HepG2 (A) and HuH7 (B) cells. After adenovirus-mediated overexpression of each gene, protein samples were prepared from cells at 0, 24, 48, 72, and 96 h, and were then subjected to western blotting using the indicated antibodies (left). Actin was used as an internal control. (C–H) Flow cytometry analysis of mCherry fluorescent protein, which was used as marker protein. Negative control cells (C) and adenovirus-infected cells, which were respectively infected with adenovirus carrying LacZ (D), PRKAG2 (E), TGFBR2 (F), and EXT1 (G), were used to analyze the ratio of mCheery positive cells. Gate was created by analysis of forward scatter (FS) and side scatter (SS) (each left panel). Percent histogram was determined by analysis of mCherry fluorescence intensity (each right panel). (H) Overlaid histogram shown in Figure S2C–S2H.
(TIF)

Figure S3 **Increment of 5-FU- and IFN-α/5-FU-induced nuclear fragmentation by TGFBR2 and EXT1.** Nuclear fragmentation shown in Figure 3A was counted and was normalized to total cell number.
(TIF)

Figure S4 **Effect of TGFBR2 on Smad-independent pathway.** JNK, p38 MAPK, and their phosphorylation were examined in HepG2 and HuH7 cells. After overexpression of each gene, cells were treated with indicated concentration of 5-FU and IFN-α for 48 h. Total JNK and p38 was used as an internal control.
(TIF)

Figure S5 **Correlation between gene expressions and survival period of HCC patients with or without HCV antibody.** (A–C) Gene expression levels in responders and non-responders to IFN-α/5-FU therapy. *PRKAG2* (A), *TGFBR2* (B), and *EXT1* (C) expression levels in clinical HCC patients. mRNA expression levels were normalized to β-actin. Statistical significance was determined by the Mann-Whitney *U* test. *P<0.05. (D) Correlation of *EXT1* expression levels with the survival periods. Data were analyzed by the Spearman’s rank correlation method. (E) Survival rates of the HCV-positive and HCV-negative patients treated with IFN-α/5-FU therapy. Data were analyzed by the Spearman’s rank correlation test. (F) *TGFBR2* mRNA expression levels in HCV-positive and HCV-negative patients. Statistical significance was determined by the Mann-Whitney *U* test. *P<0.05.
(TIF)

Table S1 **Primers used in the experiments.** All used in this study were obtained in purified form. Rz1 - Rz6 were used for the construction of random ribozyme library as described in Figure S1. N indicates any nucleotide (A, G, C and T). PRKAG2 *Bam*HI - attB2 reverse were used for the construction of adenovirus plasmid DNA carrying PRKAG2, TGFBR2 and EXT1.
(DOC)

Table S2 **Results of BLAST search of original ribozyme library and plasmid DNAs recovered after ten cycles of screening.** The ribozyme target recognition sequences recovered from one hundred colonies of *E. coli* transformed by original ribozyme library, and plasmid DNAs recovered after ten cycles of screening were analyzed by using the BLAST. The numbers of colonies, whose sequences target the same gene, were shown.
(DOC)

Table S3 **Univariate analysis of factors associated with outcome.** Statistical analysis was performed using log rank test. *P<0.05. Each parameter was divided into two categories according to the median line.
(DOC)

File S1 **Supporting Materials and Methods used in the experiments.**
(DOC)
