# Supplementary figures and images for: Identification of the Genes Chemosensitizing Hepatocellular Carcinoma Cells to Interferon-α/5-Fluorouracil and Their Clinical Significance
Source: PLoS One. 2013 Feb 15;8(2):e56197. doi: 10.1371/journal.pone.0056197 (PMC3574150; doi:10.1371/journal.pone.0056197)

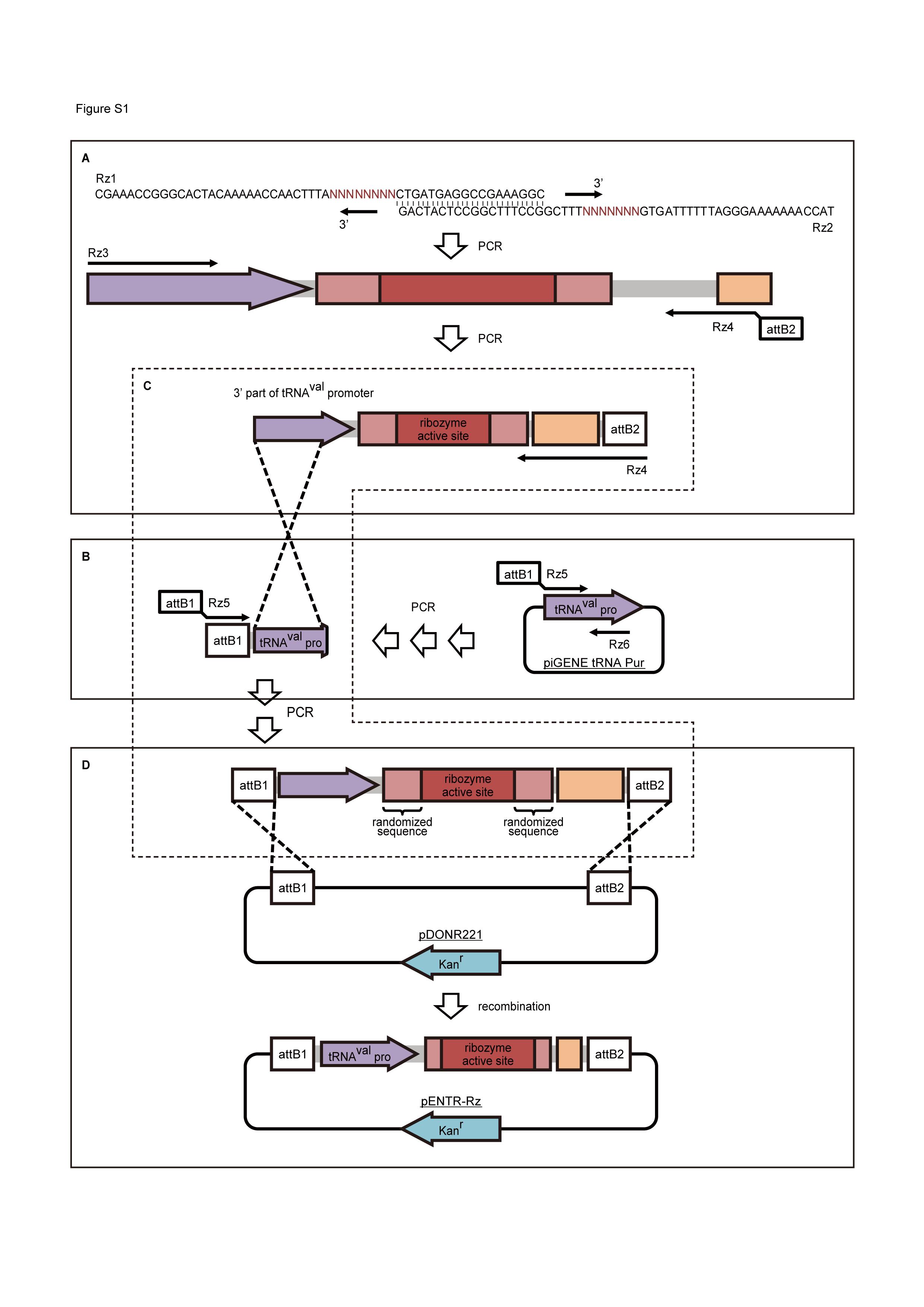

Supplement: Figure S1 — Scheme for construction of ribozyme library. A plasmid DNA (pDNA) library expressing random ribozyme genes with as large as about 6×10^6^ target recognition sequences from synthesized oligonucleotides (Rz1–Rz6; the sequences were listed in Table S1) using PCR and the Gateway technologies. (TIF) [file pone.0056197.s001.tif]

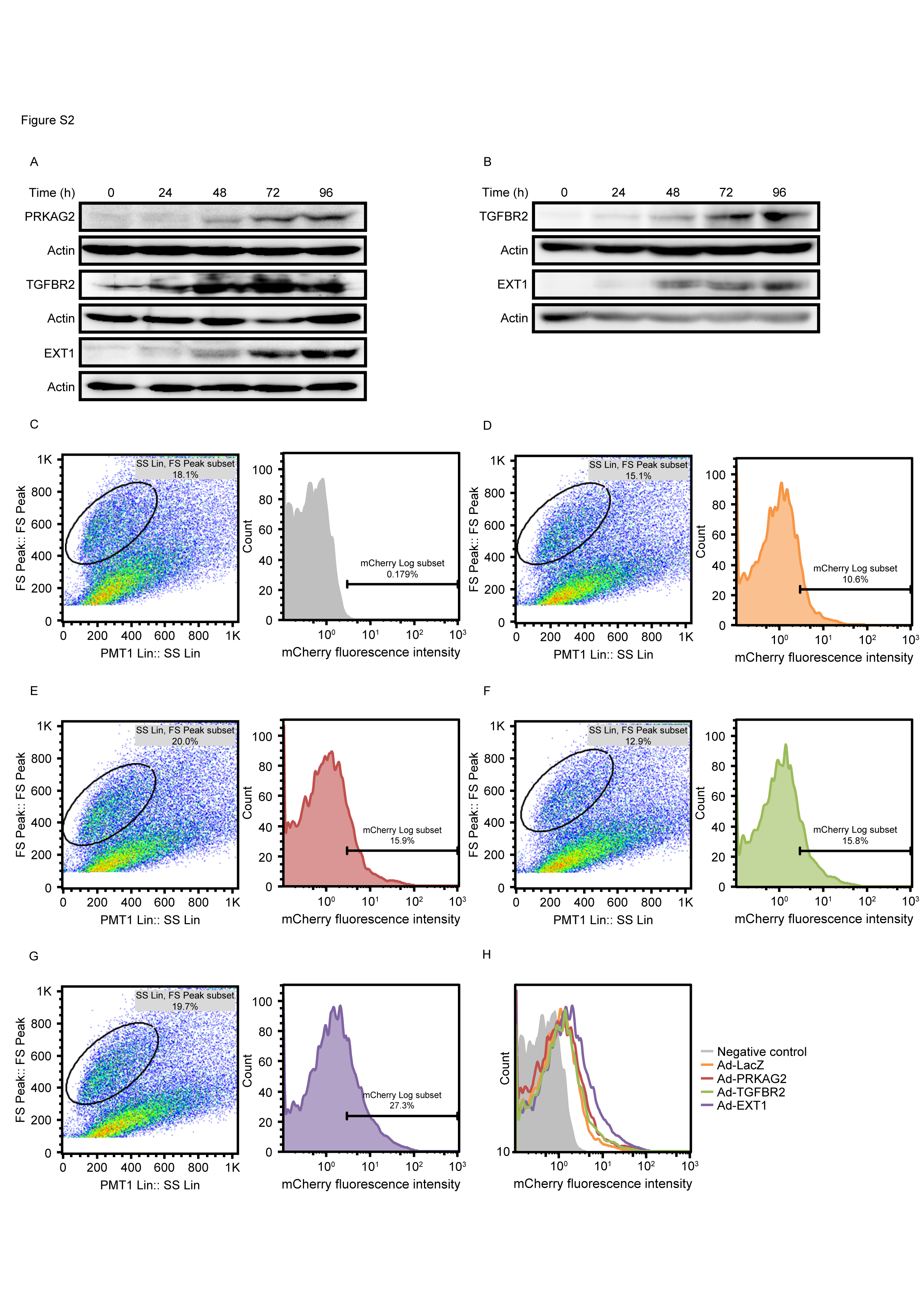

Supplement: Figure S2 — Detection of adenovirus-mediated gene transfer. These protein expressions after adenovirus-mediated gene transfer were examined in HepG2 (A) and HuH7 (B) cells. After adenovirus-mediated overexpression of each gene, protein samples were prepared from cells at 0, 24, 48, 72, and 96 h, and were then subjected to western blotting using the indicated antibodies (left). Actin was used as an internal control. (C–H) Flow cytometry analysis of mCherry fluorescent protein, which was used as marker protein. Negative control cells (C) and adenovirus-infected cells, which were respectively infected with adenovirus carrying LacZ (D), PRKAG2 (E), TGFBR2 (F), and EXT1 (G), were used to analyze the ratio of mCheery positive cells. Gate was created by analysis of forward scatter (FS) and side scatter (SS) (each left panel). Percent histogram was determined by analysis of mCherry fluorescence intensity (each right panel). (H) Overlaid histogram shown in Figure S2C–S2H. (TIF) [file pone.0056197.s002.tif]

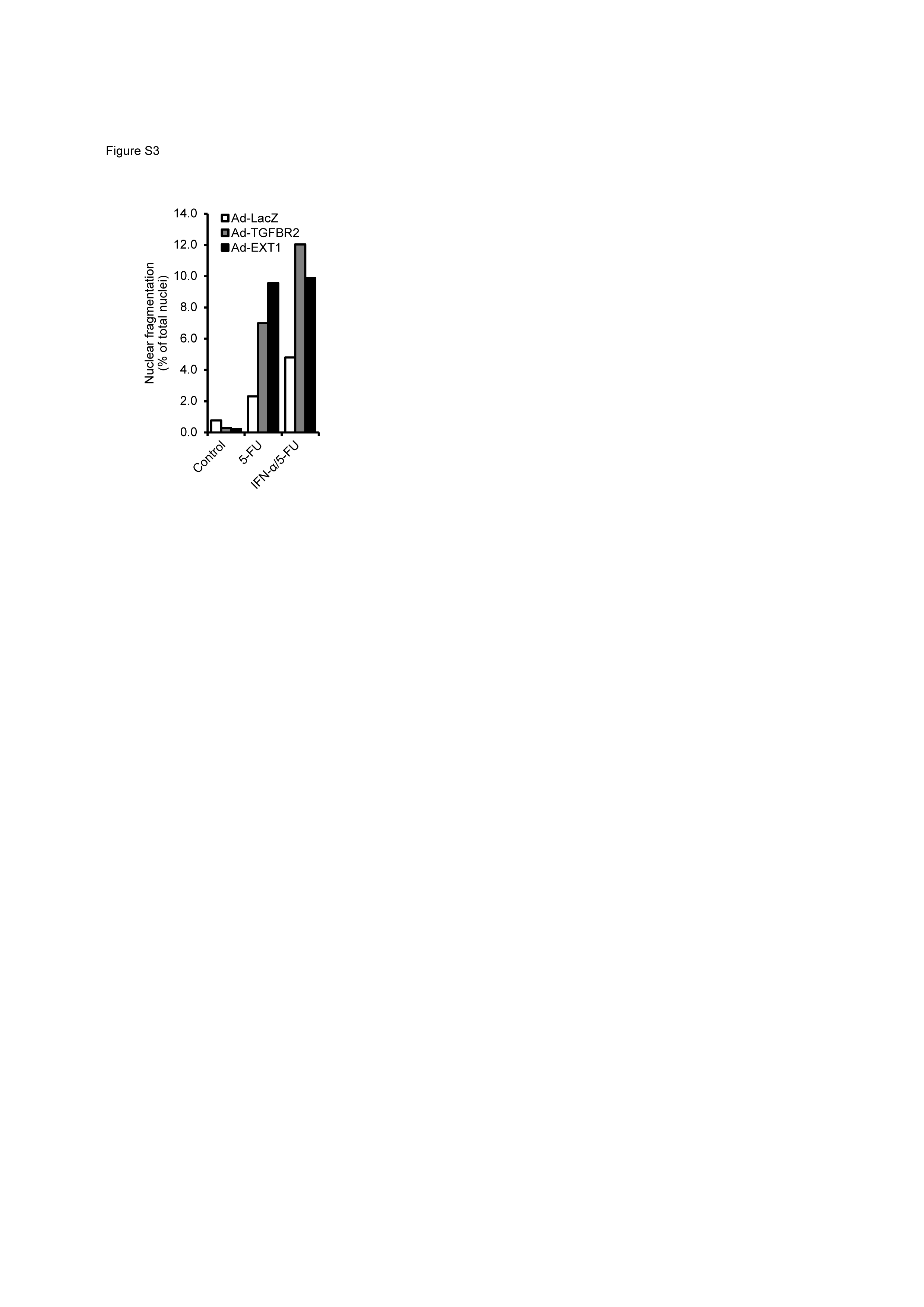

Supplement: Figure S3 — Increment of 5-FU- and IFN-α/5-FU-induced nuclear fragmentation by TGFBR2 and EXT1. Nuclear fragmentation shown in Figure 3A was counted and was normalized to total cell number. (TIF) [file pone.0056197.s003.tif]

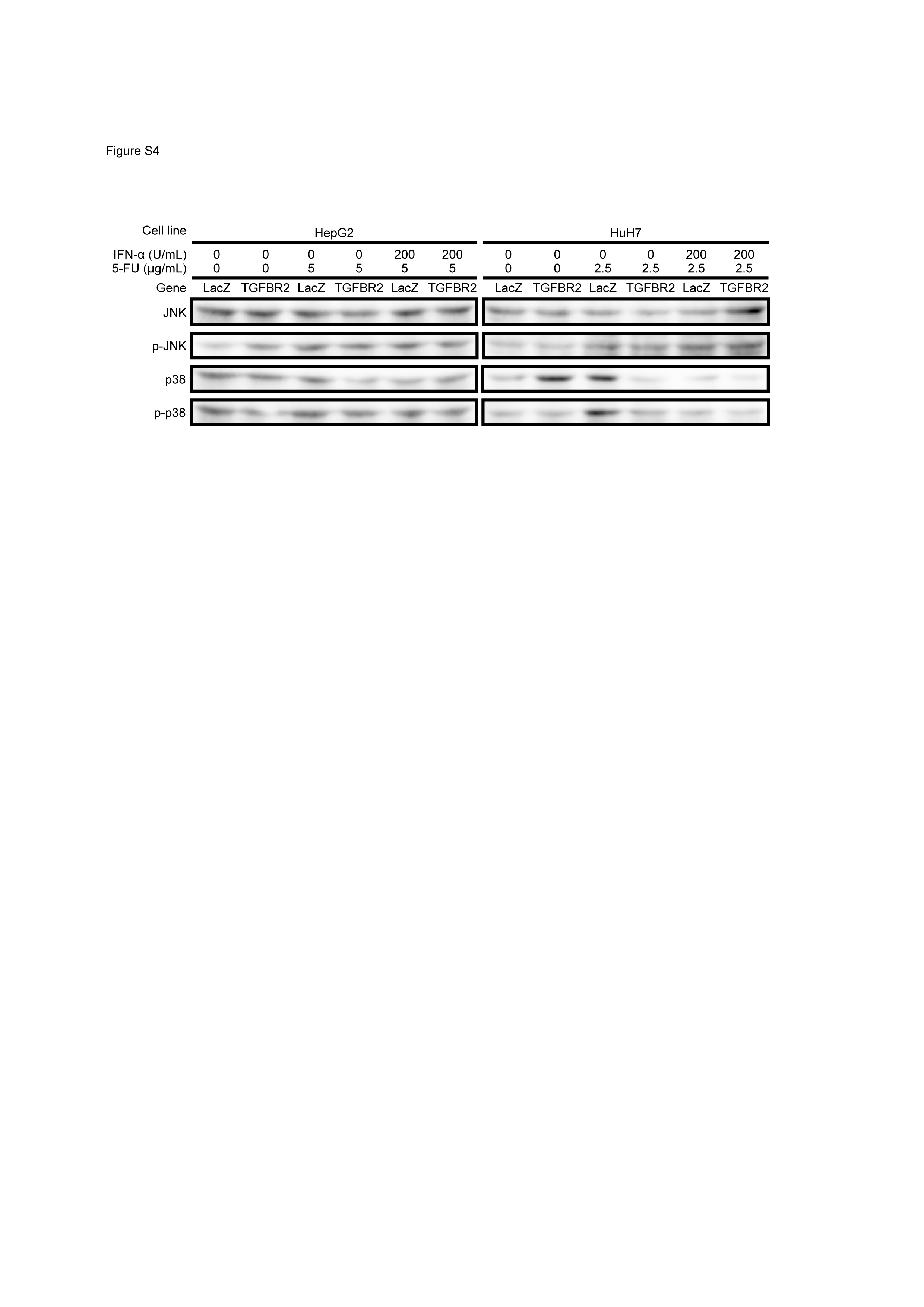

Supplement: Figure S4 — Effect of TGFBR2 on Smad-independent pathway. JNK, p38 MAPK, and their phosphorylation were examined in HepG2 and HuH7 cells. After overexpression of each gene, cells were treated with indicated concentration of 5-FU and IFN-α for 48 h. Total JNK and p38 was used as an internal control. (TIF) [file pone.0056197.s004.tif]

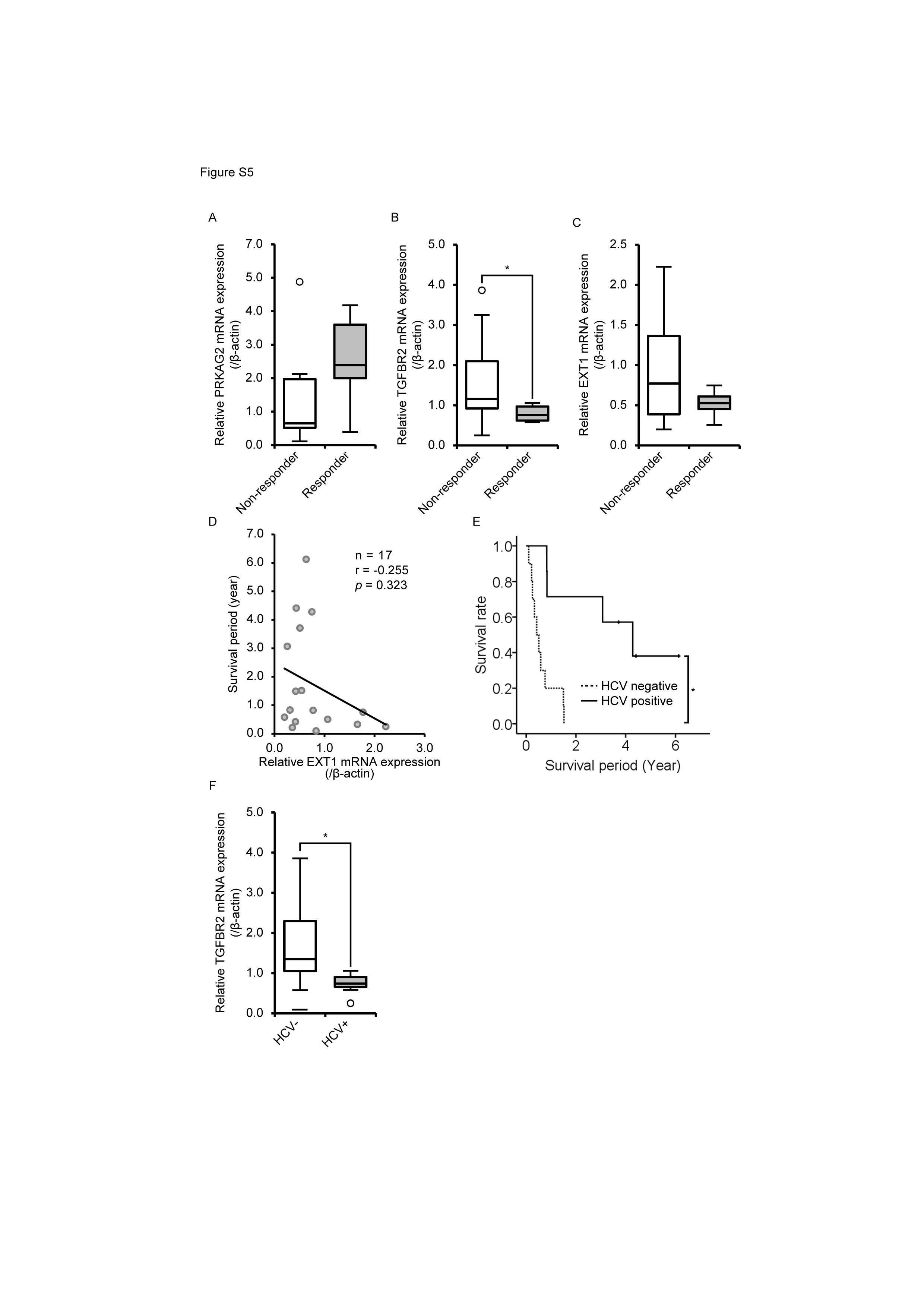

Supplement: Figure S5 — Correlation between gene expressions and survival period of HCC patients with or without HCV antibody. (A–C) Gene expression levels in responders and non-responders to IFN-α/5-FU therapy. PRKAG2 (A), TGFBR2 (B), and EXT1 (C) expression levels in clinical HCC patients. mRNA expression levels were normalized to β-actin. Statistical significance was determined by the Mann-Whitney U test. *P<0.05. (D) Correlation of EXT1 expression levels with the survival periods. Data were analyzed by the Spearman’s rank correlation method. (E) Survival rates of the HCV-positive and HCV-negative patients treated with IFN-α/5-FU therapy. Data were analyzed by the Spearman’s rank correlation test. (F) TGFBR2 mRNA expression levels in HCV-positive and HCV-negative patients. Statistical significance was determined by the Mann-Whitney U test. *P<0.05. (TIF) [file pone.0056197.s005.tif]
