# Supplementary material for: Identification of the Genes Chemosensitizing Hepatocellular Carcinoma Cells to Interferon-α/5-Fluorouracil and Their Clinical Significance
Source: PLoS One. 2013 Feb 15;8(2):e56197. doi: 10.1371/journal.pone.0056197 (PMC3574150; doi:10.1371/journal.pone.0056197)
Supplement: Table S1 — Primers used in the experiments. All used in this study were obtained in purified form. Rz1 - Rz6 were used for the construction of random ribozyme library as described in Figure S1. N indicates any nucleotide (A, G, C and T). PRKAG2 BamHI - attB2 reverse were used for the construction of adenovirus plasmid DNA carrying PRKAG2, TGFBR2 and EXT1. (DOC) [file pone.0056197.s006.doc]

Table S1. Primers used in the experiments

| Primer name | Sequence |
| --- | --- |
| Rz1 | 5'-CGAAACCGGGCACTACAAAAACCAACTTTANNNNNNNNCTGATGAGGCCGAAAGGC-3' |
| Rz2 | 5'-TACCAAAAAAAGGGATTTTTTAGTGNNNNNNNTTTCGGCCTTTCGGCCTCATCAG-3' |
| Rz3 | 5'-CGAAACCGGGCACTACAAAAAC-3' |
| Rz4 | 5'-GGGGACCACTTTGTACAAGAAAGCTGGGTATGGTACCAAAAAAAGGGATTTTTTAGTG-3' |
| Rz5 | 5'-GGGGACAAGTTTGTACAAAAAAGCAGGCTTGTAACCGTTGGTTTCCGTAGTGTA-3' |
| Rz6 | 5'-TGGTTTTTGTAGTGCCCGGTTT-3' |
| attB1 forward | 5'-GGGGACAAGTTTGTACAAAAAAGCAGGCTTCCCGGACTCAGATCTCGAGCTCAAGC-3' |
| attB2 reverse | 5'-GGGGACCACTTTGTACAAGAAAGCTGGGTCTGGTATGGCTGATTATGATCTAGAG-3' |
| PRKAG2 forward | 5'-ACGAAGCAGTAGAAGACTCAGAAA-3' |
| PRKAG2 reverse | 5'-CGTTGGCTACCAAAGCAAAGAAG-3' |
| TGFBR2 forward | 5'-GCAGGTGGGAACTGCAAGAT-3' |
| TGFBR2 reverse | 5'-GAAGGACTCAACATTCTCCAAATTC-3' |
| FOXP2 forward | 5'-CCAATCGCTGCCTCAAG-3' |
| FOXP2 reverse | 5'-AGTGTTGGAGGAGGTAGTCG-3' |
| EXT1 forward | 5'-GCTCTTGTCTCGCCCTTTTGT-3' |
| EXT1 reverse | 5'-TGGTGCAAGCCATTCCTACC-3' |
| POLR2J4 forward | 5'-AGATGAGGTGCGAACAAAGC-3' |
| POLR2J4 reverse | 5'-CCCTGCACGTCTCTCCTAC-3' |
| b-actin forward | 5'-CACTCTTCCAGCCTTCCTTCC-3' |
| b-actin reverse | 5'-CGTACAGGTCTTTGCGGATGTC-3' |
| CHOP forward | 5'-AAGATGAGCGGGTGGCA-3' |
| CHOP reverse | 5'-TGCTTTCAGGTGTGGTGATGT-3' |
| BiP/GRP78 forward | 5'-CGTGGAATGACCCGTCTG-3' |
| BiP/GRP78 reverse | 5'-CCACCTCCAATATCAACTTGAATGTATG-3’ |
| TGFB1 forward | 5'-TTTTGATGTCACCGGAGTTG-3' |
| TGFB1 reverse | 5'-AACCCGTTGATGTCCACTTG-3' |
| PRKAG2 *Bam*HI | 5’-CCGGGATCCACGGCGGTCACTCCGTTTCTG-3’ |
| PRKAG2 *Eco*RI | 5’-CGCGAATTCGGGAGGGAAGGAGGGGACC-3’ |
| TGFBR2 *Bam*HI | 5'-CCGGGATCCTCGGTCTATGACGAGCAGCG-3' |
| TGFBR2 *Eco*RI | 5'-CCGGAATTCAGCCTGCCCCAGAAGAGATA-3' |
| EXT1 *Bam*HI | 5'-CGCGGATCCGGCAGGACACATGCAGGCCAAA-3' |
| EXT1 *Eco*RI | 5'-CCGGAATTCCTCAGCCGGATTCCACAAAGTC-3' |

All used in this study were obtained in purified form. Rz1 - Rz6 were used for the construction of random ribozyme library as described in Figure S1. N indicates any nucleotide (A, G, C and T). PRKAG2 *Bam*HI - attB2 reverse were used for the construction of adenovirus plasmid DNA carrying PRKAG2, TGFBR2 and EXT1.
