# Supplementary material for: Identification of the Genes Chemosensitizing Hepatocellular Carcinoma Cells to Interferon-α/5-Fluorouracil and Their Clinical Significance
Source: PLoS One. 2013 Feb 15;8(2):e56197. doi: 10.1371/journal.pone.0056197 (PMC3574150; doi:10.1371/journal.pone.0056197)
Supplement: Table S2 — Results of BLAST search of original ribozyme library and plasmid DNAs recovered after ten cycles of screening. The ribozyme target recognition sequences recovered from one hundred colonies of E. coli transformed by original ribozyme library, and plasmid DNAs recovered after ten cycles of screening were analyzed by using the BLAST. The numbers of colonies, whose sequences target the same gene, were shown. (DOC) [file pone.0056197.s007.doc]

Table S2. Results of BLAST search of original ribozyme library and plasmid DNAs recovered after ten cycles of screening

| Ribozyme-targeted gene | Gene symbol | Number of ribozyme which target each gene (Rz-C0) | Number of ribozyme which target each gene (Rz-C10) |
| --- | --- | --- | --- |
| protein kinase, AMP-activated, gamma 2 non-catalytic subunit | PRKAG2 | 0 | 8 |
| transforming growth factor, beta receptor type II (70/80kDa) | TGFBR2 | 0 | 7 |
| forkhead box P2 | FOXP2 | 0 | 6 |
| exostoses (multiple) 1 | EXT1 | 0 | 4 |
| polymerase (RNA) II (DNA directed) polypeptide J4, pseudogene | POLR2J4 | 0 | 4 |

The ribozyme target recognition sequences recovered from one hundred colonies of *E. coli* transformed by original ribozyme library, and plasmid DNAs recovered after ten cycles of screening were analyzed by using the BLAST. The numbers of colonies, whose sequences target the same gene, were shown.
