# Supplementary material for: Identification of the Genes Chemosensitizing Hepatocellular Carcinoma Cells to Interferon-α/5-Fluorouracil and Their Clinical Significance
Source: PLoS One. 2013 Feb 15;8(2):e56197. doi: 10.1371/journal.pone.0056197 (PMC3574150; doi:10.1371/journal.pone.0056197)
Supplement: Table S3 — Univariate analysis of factors associated with outcome. Statistical analysis was performed using log rank test. *P<0.05. Each parameter was divided into two categories according to the median line. (DOC) [file pone.0056197.s008.doc]

Table S3. Univariate analysis of factors associated with outcome

|  | Category | *P*-value |
| --- | --- | --- |
| Age (years) | ≥65 vs. <65 | 0.2023 |
| Gender | Male vs. female | 0.8778 |
| Child-pugh | A vs. B | 0.5302 |
| HBs Ag | Presence vs. absence | 0.0975 |
| HCV Ab | Presence vs. absence | *0.0010** |
| PRKAG2 expression | ≥1.89 vs. <1.89 | *0.0358** |
| TGFBR2 expression | ≥1.03 vs. <1.03 | 0.0837 |
| EXT1 expression | ≥0.54 vs. <0.54 | 0.5149 |

Statistical analysis was performed using log rank test. **P* < 0.05. Each parameter was divided into two categories according to the median line.
