# Supplementary material for: Identification of the Genes Chemosensitizing Hepatocellular Carcinoma Cells to Interferon-α/5-Fluorouracil and Their Clinical Significance
Source: PLoS One. 2013 Feb 15;8(2):e56197. doi: 10.1371/journal.pone.0056197 (PMC3574150; doi:10.1371/journal.pone.0056197)
Supplement: File S1 — Supporting Materials and Methods used in the experiments. (DOC) [file pone.0056197.s009.doc]

**Supporting Information**

**Identification of the genes chemosensitizing hepatocellular carcinoma cells to interferon-α/5-fluorouracil and their clinical significance**

Tomohiko Sakabe^1^, Hiroyuki Tsuchiya^2^, Keita Kanki^1^, Junya Azumi^1^, Kazue Gonda^1^, Yusuke Mizuta^1^, Daisaku Yamada^3^, Hiroshi Wada^3^, Kohei Shomori^4^, Hiroaki Nagano^3^, Goshi Shiota^1^

Contact Information: Goshi Shiota, MD, PhD,

Division of Molecular and Genetic Medicine, Department of Genetic Medicine and Regenerative Therapeutics, Graduate School of Medicine, Tottori University, Yonago 683-8503, Japan

Tel.: +81-859-38-6431; Fax: +81-859-38-6430; E-mail: [gshiota@med.tottori-u.ac.jp](mailto:gshiota@med.tottori-u.ac.jp)

**Supporting Materials and Methods**

**Construction of ribozyme library**

Steps refer to those in Figure S1.

Step A: Amplification of ribozyme gene

Ribozyme genes containing random target recognition sequences (in red: N indicates any nucleotide) were double-stranded by PCR using KOD plus (TOYOBO, Osaka, Japan) with Rz1 and Rz2 oligonucleotides (Table S1). The double-stranded ribozyme genes were further amplified by PCR using Rz3 and Rz4 primers. The products contain 3’-part of a valine tRNA promoter (tRNA^val^ pro; purple), ribozyme active site (dark red), randomized target recognition sequences (red) and transcription terminator sequence (orange), and were attached an attB2 recombination sequence (white) at its 3’-terminus.

Step B: Preparation of tRNA^val^ promoter with an attB1 recombination sequence

piGENE tRNA Pur (iGENE Therapeutics, Inc, Tokyo, Japan) was used as a template for PCR using Rz5 and Rz6 to obtain tRNA^val^ promoter fragment conjugated with an attB1 recombination sequence at 5’-teminus (Table S1).

Step C: Preparation of random ribozyme expression cassette flanked by attB recombination sequences

PCR products generated in steps A and B shared a homologous sequence in their 5’ and 3’ regions, respectively, so that PCR using these PCR products as templates with Rz4 and Rz5 primers resulted in an amplification of larger PCR products by conjugation of them (Table S1). The PCR produced the tRNA^val^ promoter-driven random ribozyme expression cassette flanked by the attB recombination sequences.

Step D: Recombination of the random ribozyme expression cassette into plasmid DNA by the Gateway technology

The PCR product obtained in step C was inserted into pDONR221 (Invitrogen) containing attP sites using the Gateway technology, which allows the direct insertion of a gene of interest into a plasmid DNA by homologous recombination between attB and attP sites (BP reaction). This reaction produced tRNA^val^ promoter-driven random ribozyme expression plasmid DNA (pENTR-Rz). The resultant pENTR-Rz were propagated by electroporation into DH5α *E. coli* in a condition of 2,500 V, 25 μF and 201 kΩ (Gene Pulser System, Bio-Rad Japan, Tokyo, Japan). This step was repeated until 5,962,500 of *E.coli* colonies were obtained.

The diversity of the library was estimated as follows; One hundred sequences of ribozyme genes obtained from independent *E.coli* clones were analyzed, and compared each other to find out the plasmid DNAs with the same target recognition sequence. As a result, we found no overlap in this analysis, suggesting that the provability of the appearance of an overlapping sequence is less than 0.01. Multiplying 5,962,500 with (1 - 0.01) resulted in 5,902,875 sequences of the random ribozyme library obtained.

**Screening for genes involved in the efficacy of IFN-α/5-FU combination therapy**

HepG2 cells transfected with the ribozyme expression plasmid library were treated with 5 μg/mL 5-FU for 72 h. The surviving adherent cells were harvested and a library was isolated from these cells. This library was amplified by transformation into DH5α *Escherichia coli* and then transfected into HepG2 cells again. This cycle was repeated 10 times in order to eliminate cells expressing a ribozyme that targets chemosensitivity-unrelated genes and successfully concentrate the ribozymes rendering HepG2 cells resistant to 5-FU. Moreover, 100 target ribozyme recognition sequences that were recovered before screening and after 10 cycles of screening were subjected to a BLAST search to retrieve genes with homologous sequences. Finally, we ranked the retrieved genes according to the numbers of ribozymes overlapping a transcript.

**Hoechst staining**

We treated HepG2 cells, which were infected with adenoviruses carrying *TGFBR2*, *EXT1*, or control gene (*LacZ*), with 5 μg/mL of 5-FU with or without 200 U/mL of IFN-α for 48 h. The cells were then further incubated with 10 μM Hoechst 33258 (Sigma-Aldrich, St. Louis, MO, USA) for 1 h.

**Quantification of caspase-3/7 activity**

After adenoviral infection, the cells were further incubated with 5-FU in the presence or absence of IFN-α. For caspase-3/7 activity measurement, we used the Caspase-Glo 3/7 Assay Kit (Promega, Madison, WI, USA) according to the manufacturer’s protocol.

**Luciferase reporter assay**

Cells were co-transfected with the TGF-β-responsive reporter plasmid p3TP-Lux (Addgene, Cambridge, USA) containing TGF-β responsive elements [1] and phRL-TK (Promega) as an internal control by using the FuGENE 6 reagent (Roche Applied Science). At 24 h after incubation, the cells were infected with adenoviruses carrying each gene. The cells were treated with 5 (HepG2) or 2.5 (HuH7) μg/mL 5-FU in the presence or absence of 200 U/mL IFN-α for 48 h. Luciferase activity was measured using the Dual-Luciferase Reporter Assay Kit (Promega) according to the manufacturer’s instructions.

**Immunohistochemistry**

Immunohistochemistry was performed on the 3 μm formalin-fixed, paraffin-embedded tissue sections using the Avidin Biotin staining technique. After the deparaffinization step, sections were treated with 3% hydrogen peroxide solution to eliminate endogenous peroxidase activity. Subsequently, sections microwave treated in sodium citrate buffer to improve the antigen retrieval. Stains were performed using the antibodies against PRKAG2 (Cell Signaling Technology), TGFBR2 (Santa Cruz Biotechnology) and VECTASTAIN Elite ABC Kit (Vector Laboratories, Burlingame, USA) according to the manufacturer’s instructions. Color development was achieved by the treatment with ImmPACT DAB Substrate (Vector Laboratories).

**Supporting Information References**

1. Wrana JL, Attisano L, Cárcamo J, Zentella A, Doody J, et al. (1992) TGF beta signals through a heteromeric protein kinase receptor complex. Cell 71: 1003-1014.
